# Mechanical Fracturing of Core-Shell Undercooled Metal Particles for Heat-Free Soldering

**DOI:** 10.1038/srep21864

**Published:** 2016-02-23

**Authors:** Simge Çınar, Ian D. Tevis, Jiahao Chen, Martin Thuo

**Affiliations:** 1 Department of Materials Science & Engineering, Iowa State University, Ames, IA 50011 USA; 2 Micro-electronics Research Center, Iowa State University, Ames, IA 50011 USA; 3 Center for bio-plastics and bio-renewables, Iowa State University, Ames, IA 50011 USA

## Abstract

Phase-change materials, such as meta-stable undercooled (supercooled) liquids, have been widely recognized as a suitable route for complex fabrication and engineering. Despite comprehensive studies on the undercooling phenomenon, little progress has been made in the use of undercooled metals, primarily due to low yields and poor stability. This paper reports the use of an extension of droplet emulsion technique (SLICE) to produce undercooled core-shell particles of structure; metal/oxide shell-acetate (‘/’ = physisorbed, ‘-’ = chemisorbed), from molten Field’s metal (Bi-In-Sn) and Bi-Sn alloys. These particles exhibit stability against solidification at ambient conditions. Besides synthesis, we report the use of these undercooled metal, liquid core-shell, particles for heat free joining and manufacturing at ambient conditions. Our approach incorporates gentle etching and/or fracturing of outer oxide-acetate layers through mechanical stressing or shearing, thus initiating a cascade entailing fluid flow with concomitant deformation, combination/alloying, shaping, and solidification. This simple and low cost technique for soldering and fabrication enables formation of complex shapes and joining at the meso- and micro-scale at ambient conditions without heat or electricity.

Phase-change materials have been recognized to bear great potential. One such class of materials depends on inhibiting liquid-solid phase change below their melting point (T_m_) – so called undercooling. Undercooling is widely observed since it can be achieved by eliminating heterogeneous nucleation site(s) or solidification catalysts^1^,^2^,^3^,^4^,^5^,^6^,^7^,^8^,^9^. Undercooling of metals has been widely studied, primarily to inform metal processing and microstructure evolution during solidification^2^,^5^,^6^. Due to the metastable nature of undercooled metal particles, their production in good yields is an experimental challenge. This challenge can be overcome through; i) elimination of heterogeneous nucleating sites, or other sites with high potency for catalyzing solidification^1^,^7^,^9^,^10^, and, ii) minimizing the container effects (nucleation) by employing the droplet dispersion or containerless techniques in synthesis of undercooled particles^3^,^4^,^11^. Using these techniques, undercooling values as high as ~0.3–0.4 T_m_ have been reported^6^,^7^. One of the highest undercooling achieved so far is 0.7 T_m_ for 3–15 nm gallium particles^10^. The literature on metal undercooling, however, is heavily skewed towards studies on understanding the solidification behavior and thermodynamics of metal systems^12^,^13^,^14^,^15^,^16^,^17^,^18^,^19^,^20^,^21^,^22^,^23^ and, to the best of our knowledge, there is limited discussion on practical applications except for heat transfer^24^ and production of metastable solids^11^. A major barrier limiting application could be challenges in preparing stable undercooled particles in high yields and across size scales, especially where large undercooling values are desired^1^,^2^,^5^,^6^. In the containerless drop tube technique, for example, a particle is undercooled only during free fall. Droplet emulsion techniques, on the other hand, allow for the production of more than one particle at a time only if the carrier liquid can maintain a thin, inert surface coating inhibit solidification^25^. The undercooling phenomenon is size dependent and is much more readily attained with nanoscale particles, hence, size-driven optimization to maximize undercooling^6^,^26^,^27^,^28^.

We hypothesized that if an over-heated metal melt is encapsulated in a thin, uniform stable layer, while also reducing the size of the particle, a stable undercooled liquid nano- and/or micro- particle can be formed. The so-produced particles can then be used for manufacturing/fabrication or as a low temperature solder (through mechanical or chemical bonds). The use of undercooled metals significantly below their melting point, T_m_, eliminates many drawbacks of currently available joining or fabrication techniques^29^,^30^,^31^,^32^,^33^,^34^,^35^,^36^. We hypothesized that applying the recently reported SLICE (Shearing Liquids Into Complex ParticlEs) technique (Fig. 1A)^37^, three-layered core-shell undercooled particle (Fig. 1D) can be fabricated analogous to commonly made liquid marbles^38^,^39^,^40^. Under SLICE, a liquid or molten metal is sheared to particles of desired size with concomitant surface oxidation to give a passivating layer on which a second (organic) layer is assembled to give smooth surfaces – a key component in efficient particle assembly^37^. If metals with low melting temperature are used in their undercooled state, or with significant undercooling, then manufacturing and joining can be performed even at room temperature. Main benefits of the proposed approach are; i) reduces the need for advanced instrument, ii) limited need for skilled manpower, iii) minimizes energy needs, iv) significantly improves production efficiency, v) is low-cost and uses lead-free materials, and, vi) reduces fabrication cost.

## Results and Discussion

**Figure 1:**
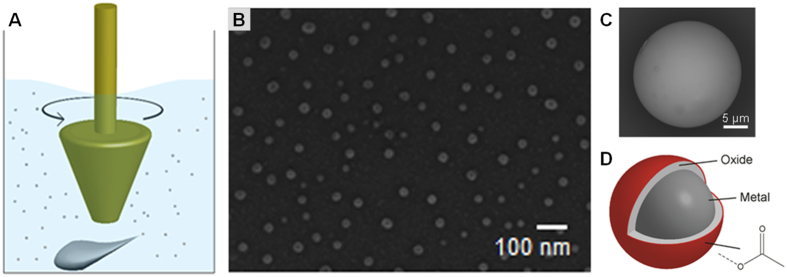
(**A**) Schematic of the SLICE process, where a rotating implement shears the liquid or molten metal into smaller pieces in an acid containing carrier fluid, (**B**) SEM micrograph of EGaIn nanoparticles derived from SLICE, (**C**) a close-up of a liquid metal microparticle, (**D**) schematic of cross-section of proposed particle structure – outer layer oxide with a chelated organic stabilizer encapsulating an undercooled liquid metal core.

**Figure 2:**
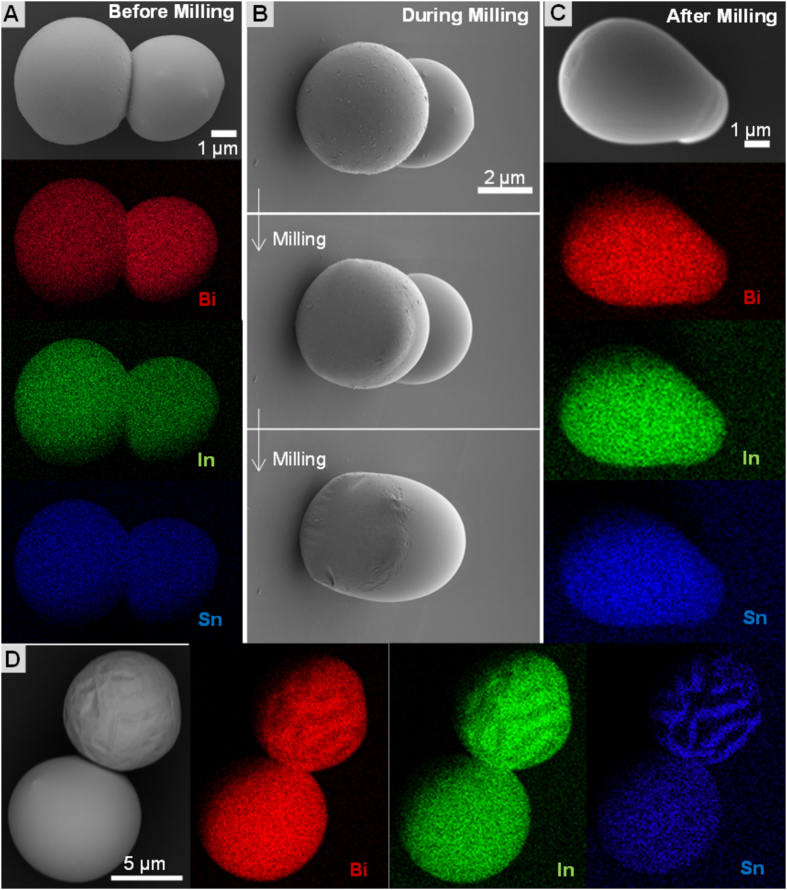
Core-shell structure enables undercooling. Field’s metal particles are in liquid state at ambient temperature. (**A**) Undercooled Field’s metal particles. The elemental EDS map shows the uniform composition. (**B**) Self-assembled particles are milled by focused ion beam until the thin oxide surface layers are removed and the liquid flows to coalescence into a non-spherical shape. (**C**) The coalesced particle shows uniform elemental composition. (**D**) The undercooled and solidified eutectic metal particles. Solidified particles show phase segregation and consist at least two different compositions as seen in the Energy-selective backscattered (EsB) detector images (top particle).

**Figure 3:**
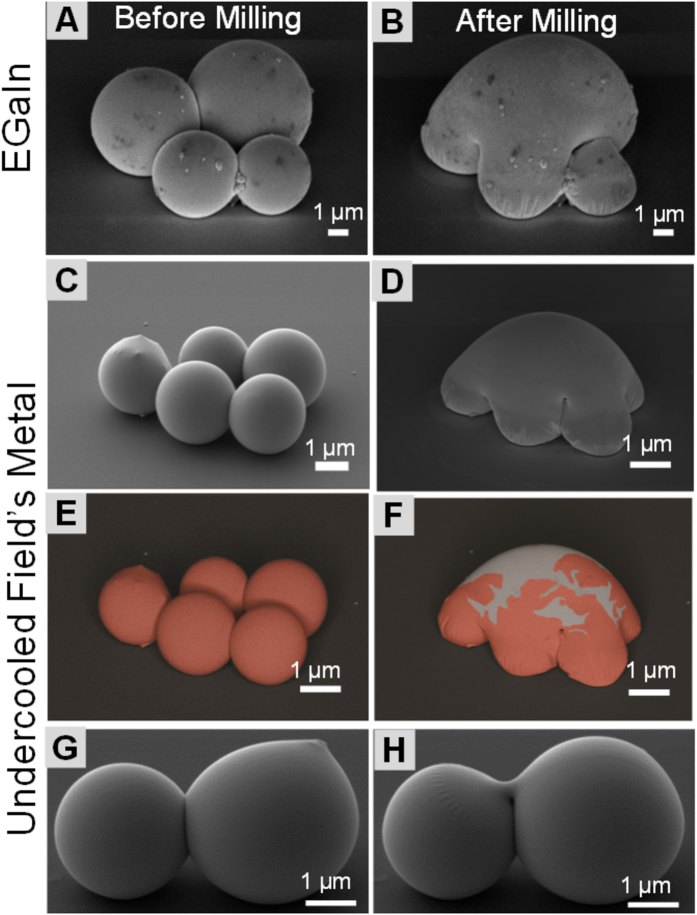
Comparison of micro- and sub-microscale sculpturing of liquid metal particles (EGaIn) at room-temperature and analogous to liquid undercooled particles (Field’s metal). Sculpturing by removal of the outer layers creates turtle-like structures from EGaIn (**A,B**) and Field’s metal (**C,D**). (**E,F**) are the false-colorized images of the EsB detector images to highlight the fractured oxide layer. (**G,H**) show the joining of two undercooled Field’s metal particles through a bridge formed by flow of undercooled metal from a hole opened by spot-milling on the particle surface using FIB. The joining in (**H**) demonstrates a form of mechanical welding whose dimensions are dependent on the size of drilled hole, viscosity of the liquid metal and solidification kinetics.

Metal droplet emulsion technique is reported as the most promising approach to attain maximum undercooling and in good yields. In the present work, we have used the SLICE technique, which involves shearing of a liquid metal or metal melt to break it to small parts with concomitant surface oxidation and functionalization (Fig. 1). We recently reported synthesis of EGaIn (eutectic gallium indium, m.p. = 15.6 °C) particles with sizes ranging from 6.4 nm to 10 μm using the SLICE technique (Fig. 1B)^37^. Here, we report the synthesis of undercooled Field’s metal (eutectic bismuth-indium-tin, m.p. = 62 °C) and eutectic Bi-Sn (m.p. = 139 °C) particles, using the SLICE technique, and demonstrate their application in heat-free mechanical soldering or in fabrication of irregularly shaped structures. Figures 2 and 3 give examples that include: *(i)* fabricating and joining complex structures formed by fracturing the oxide-acetate layer using focused ion beam (FIB) mechanical milling to create new shapes. Capillary-driven self-assembly of particles followed by removal of the outer layers by FIB, which allows the undercooled metal to flow, to form complex 3D structures that are otherwise difficult to fabricate. *(ii)* Mechanically stressing the droplets resulting in fracture of outer layers; leading to flow and subsequent coalescence of the undercooled liquid metal. Instantaneous solidification occurs due to the numerous *in situ* generated nucleation sites, in part, due to; a) oxide fragments, b) contact with substrate surface walls, c) rapid oxidation, and, d) equilibration to ambient conditions. This process leads to the formation of a strain relaxation – surface energy minimized solid structure dictated by mechanical conformity to the mold holding the undercooled particle. Manufacturing of sheet like structures, joining due to coalescence and solidification of undercooled liquid metal particles (referred to as heat-free mechanical soldering), joining of thin metal films (200 nm thick), and, healing surface defects in thin films (200 nm) using the undercooled particles are demonstrated.

In the SLICE technique^37^, removal of shear force in absence of a stabilizer leads to de-emulsification and subsequent spinodal decomposition on eutectic solidification^41^,^42^. Prevention of particle recombination, through particle architecture or coatings, leads to formation of small metal particles which could then be assembled into complex structures. The formation of a thin complex oxide-acetate outer shell layer^28^ can, potentially, eliminate nucleation sites and any adventitious solidification catalyzing entities, trapping a metal melt in the metastable liquidus state.

### Evidence for the liquid nature of the synthesized particles

Room temperature liquid metals particles, like EGaIn, have been shown to form different complex structures/shapes due to their non-Newtonian nature^25^,^43^,^44^,^45^,^46^. To demonstrate that the synthesized undercooled particles have a liquid core, we compared; i) capillary driven self-assembly and deformations at the point of contact (Fig. 2A) — even under such low mechanical stress (from capillary forces due to evaporation of H_2_O/EtOH in which the particles are re-suspended), ii) flow behavior of the core on removal of the outer shell, allowing the liquid metal to flow and recombine forming new complex structures, iii) due to the protective nature of the outer oxide shell, co-assembly of liquid undercooled and solidified eutectic alloys should be possible without the solid particle inducing solidification.

Subjecting over-heated (at least 20 °C above T_m_) Field’s metal melt to SLICE in dilute (~5%) acetic acid in diethylene glycol led to production of copious amounts of particles bearing different surface architectures. We noticed that a majority of the particles had a smooth surface texture with a few showing phase-segregation (Fig. 2, Figure S3). Figure 2A shows a dimer with homogeneously distributed constituent elements [i.e. bismuth, indium and tin as shown by energy dispersive x-ray spectroscopy (EDS)] over both particles, indicating that the encapsulated material is composed of one phase, eutectic Bi-In-Sn. Removal of the outer layers by milling with FIB leads to flow and coalescence of the particles (Fig. 2B), demonstrating that the core-content of these particles is a fluid. Having this liquid phase of Field’s metal (T_m_ = 62 °C) at room temperature, shows that the metal is undercooled. Homogeneous distribution of constituent elements in the alloy (Fig. 2C) indicates that solidification is averted probably due to lack of a nucleation sites or solidification catalysts on the surface under these high vacuum conditions. It is observed that, as expected, direct contact with a solid particle during milling of an undercooled particle with FIB results in rapid solidification since the surface of solid particle acts as a nucleation site (See supporting information Figure S4 and Movie M1).

Field’s metal solidifies into different combination of phases (possible phases include: β, γ, BiIn_2_ and/or quenched liquid) depending on the solidification conditions^41^,^42^. Witusiewicz^42^, Çadırlı^41^ and co-workers have discussed the thermodynamics and microstructures evolution of eutectic Bi-In-Sn alloys, which is beyond the scope of this study. Here, we utilize surface texture and microstructure (phase segregation) of Field’s metal to distinguish solidified eutectic particles from undercooled (meta-stable) ones. It has previously been shown that solidification of the melt leads to surface phase segregation and subsequent roughening of the metal surface^37^. Figure 2D shows solidified (striated with phase-segregation) and liquid undercooled (smooth surface and no phase-segregation) particles of Field metal next to each other.

Considering storage conditions (the undercooled particles are moved without specific precaution and stored for days at a time) and the sample preparation steps (centrifugation, filtering, vortex mixing) prior to SEM imaging, it can be inferred that the undercooled particles are fairly stable and amenable to manufacturing. As presented in Figure S3, even direct contact with a rough surface does not induce crystallization, mainly because the outer oxide-organic layer isolates the undercooled liquid from contact with the rough surfaces that are potential nucleation sites. Stability of these undercooled particles gives an opportunity to manipulate them (assemble and reconstruct by milling) analogous to the liquid metal particles. Since the solidification is possible at room temperature once the outer layer is removed, these particles could be used for manufacturing and joining of nano- and micron-size systems at ambient conditions. An inherent advantage of SLICE is that the size and polydispersity of the generated particles can be controlled through felicitous choice of the synthesis conditions. The size and polydispersity of the particles affect the nature of capillary driven assembly of these particles, hence increased complexity of structures that can be obtained.

### Fabrication of complex meso-structures through self-assembly and nanoscale milling

Having demonstrated that stable undercooled particles can be synthesized in good yields, we sought to use them to fabricate structures that are otherwise difficult to make. Milling of the oxide layer with FIB enables the fabrication, or joining, of complex structures that can either be liquid or solid at ambient conditions. Figure 2A–C demonstrates the combination of a simple dimer structure upon milling of the outer oxide-acetate layers. More complex structures can also be fabricated by combining capillary-driven self-assembly with subsequent milling as previously demonstrated with EGaIn^37^. We demonstrate that undercooled liquid metal particles can be assembled into reproducible units that upon milling gives structures that are otherwise difficult to fabricate. This idea is exemplified by fabrication of a turtle-like shapes derived, first, from an assembly of four particles of a room temperature liquid metal-EGaIn (Fig. 3A–B), then five particles of an undercooled Field’s metal melt (Fig. 3C–F) albeit of different particle size distributions. Upon solidification, these structures can be used as a multi-point electrical terminal, whose geometrical contact areas are dictated by the diameter (hence the size of the tangential contact) of assembled particles used in their fabrication. Figure 3A–D show that, as expected, similar structures can be obtained using liquid core particles irrespective of their elemental compositions or thermodynamic state^47^,^48^,^49^,^50^,^51^,^52^,^53^,^54^. Taking advantage of having at least one encapsulated liquid core particle in an assembly (in this example all particles are undercooled, but only one is sufficient to fuse all particles in the assembly), the particles could be joined/fused into a new structure. Because of the stability of the undercooled metal, the Field’s metal core–shell particles could be used analogous to liquid metals. Moreover, rather than milling small areas as in Fig. 3A–D, a hole can be made, by milling of a single point on the surface of an undercooled particles, subsequent flow of the liquid metal connects two particle by forming a small bridge (100s nm – μm) between them (Fig. 3G). This approach to joining particles can be extended to any size scale since it exploits the stabilization of the undercooled metal core and its subsequent solidification, to join two materials.

Similarity of the final products fabricated from two different materials, EGaIn and Field’s metal, not only demonstrates the considerable reproducibility of the method, but also offers an opportunity to tune mechanical properties of the final product through material selection. While EGaIn, as a room temperature liquid, has low ultimate failure strain, ε^u^, solidified Field’s metal is stiffer and can therefore bear a significantly larger mechanical stress^38^,^39^,^55^,^56^,^57^,^58^. Since the particle size can be readily varied, from few nanometers to micron sizes, by altering the shearing conditions^37^, this method can be applied over a wide range of size scales, and as such, is promising for manufacturing and joining. Turtle-like structures and the bridged dimers (Fig. 3A–H), for example, could be good candidates as a two (Field’s metal, Fig. 3H), four (EGaIn, Fig. 3A), or, five (Field’s metal, Fig. 3D) point electrical contacts with tunable contact sizes, chemical, and, mechanical properties. Change of shearing fluid to ones with higher boiling point (e.g. Polyphenyl ether pump fluid, boiling point, b.p. ≈ 750 K, or thermally stable liquids like ionic liquids) allows the extrapolation of the method to a relatively wider range of metals and alloys.

### Heat-Free Micro-soldering under Controlled Environments

**Figure 4:**
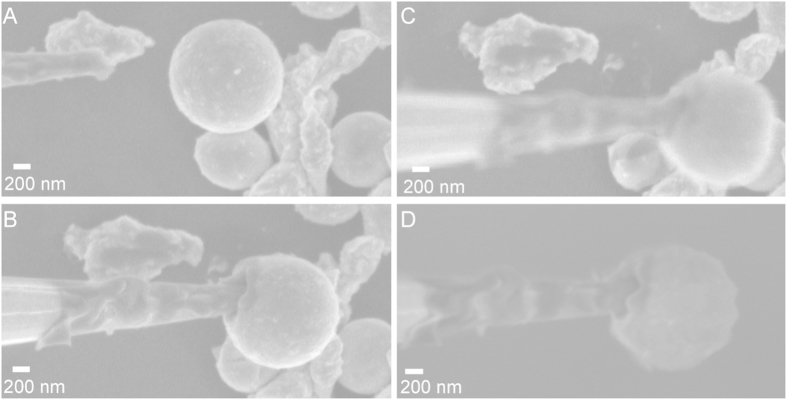
Utilization of a tungsten microprobe to penetrate and solidify an undercooled Field’s metal particle. (**A**) A probe and particle before interacting. (**B**) As the probe pushes into the particle the thin oxide shell flexes. (**C**) Probe punctures oxide shell and the liquid metal wets the probe. (**D**) The probe is lifted out of the particle, deforming it and then solidifying it. (**E**) After the probe is removed the particle stays in its deformed shape because it was solidified. (**F**) The probe was used to push the particle to the right to demonstrate that it is now solid.

Micro- or nanoscale soldering is important in various fields and for high-level fabrication^34^,^59^,^60^,^61^,^62^,^63^,^64^. To illustrate that the particles can be used for *in situ* modification or fabrication, undercooled particles were broken with a tungsten probe in the SEM, while imaging. Figure 4 (and supporting information Movie M2) shows a series of images during the breakage of an undercooled particle with an SEM omniprobe. First, attempts to rapidly break the oxide layer led to deformation of the particle indicating that the outer layer is at least elastic (Fig. 4B). When the outer layer was eventually broken, the probe wets with the liquid metal and is immediately removed, orthogonal to the approaching direction, before the particle can solidify (Fig. 4C,D). This probe retraction leads to deformation of the particle and hardening- that is, drastic increase in the Young’s modulus such that it cannot be deformed by the tip (Fig. 4E,F). When the probe is used to break a second liquid core particle without rapid withdrawal upon breakage of the outer layer, upon solidification, the tip is permanently modified (see supporting information Figure S1 and Movie M2).

### Macrofabrication

In addition to micro- and sub-microscale sculpturing described above using advanced tools and fabrication techniques, the undercooled particles can be deployed with limited technology and at room temperatures. In lieu of FIB-based milling, the outer layer can also be fractured by macroscale mechanical stressing/deformation, that is, pressing the particles to induce flow followed by coalescence and solidification. Macro-scale disk-like structure are obtained after squishing the particles with a glass slide (Supporting information Figure S2). Microstructure, as seen in the EsB detector image (Supporting information Figure S2A) and confirmed with an EDS map (Supporting information Figure S2D), shows phase-segregation analogous to those of the solid eutectic^41^ indicating that the particles have solidified analogous to the eutectic melt. A similar process is applied to two adjacent assemblies of undercooled particles to give fused micro-disk dimers (Supporting information Figure S2B) albeit with a thin boundary layer that could be due to trapped impurities or incomplete fusion of the flowing undercooled metal. We demonstrate that this method can be applied to make large flat areas (Supporting information Figure S2C, approx. 250 μm × 250 μm in this specific example), hence, sheet-like materials or thin films can also be fabricated using these undercooled particles. If the particles are tightly packed in the assembly or molds are used to aid in compacting the liquid, then the method can be used in heat-free molding. Being able to manually smash many particles at the same time to give the same microstructure (Supporting information Figure S2C) shows that almost all of the synthesized particles are undercooled, or at least in some form of deformable meta-stable state, with a yield close to unity (i.e. quantitative yield of undercooled particles). The eutectic-like microstructures and evenly distributed constituent element (Supporting information Figure S2D) would result in near isotropic chemical and mechanical properties in the fabricated products analogous to the heat-processed bulk material.

### Joining and Defect Healing: Heat-Free Mechanical Soldering (HFMS)

**Figure 5:**
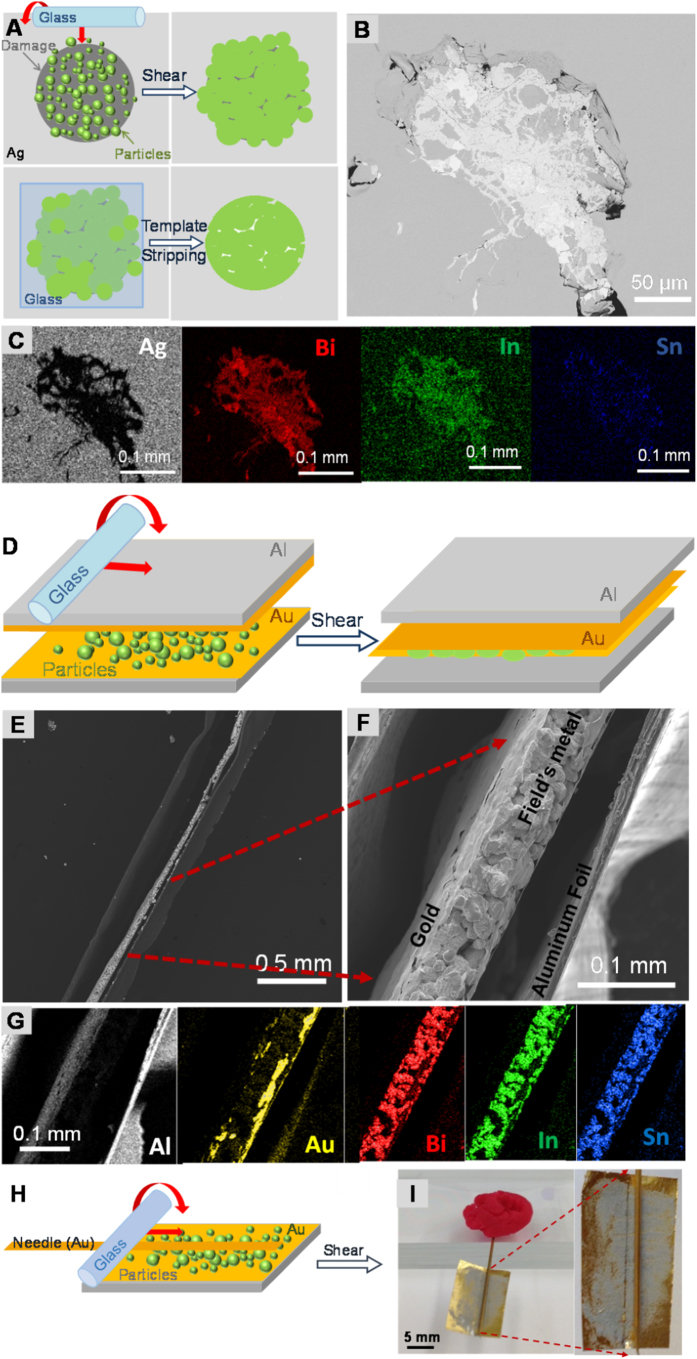
Macroscale application of undercooled particles in healing of damaged thin film (**A–C**) and joining of flat and non-flat surfaces (**D–G**). (**A**) Experimental procedure for healing a damaged silver surface. Undercooled particles were placed on a damaged area, sheared using glass cylinder, then template stripped to obtain a flat surface. (**B**) EsB detector images of a healed silver surface. (**C**) Elemental EDS map shows that Field’s metal almost fully recovered the damaged area. (**D**) Experimental procedure for joining gold films. Shearing of undercooled particles sandwiched between aluminum foil joined the thin gold coating on aluminum foil while delaminating the aluminum foil. (**E,F**) Low and high magnification images shows the Field’s metal particles can join the gold sheets while delaminated aluminum foil. (**G**) The Elemental EDS maps shows the distribution of materials in (**F**). Since the detector is at top right, left side of the gold film stayed in shadowed area, thus appeared in black even though its existence is evident in (**F**). (**H**) To illustrate ability to solder non-flat surfaces, Au coated wire is adhered to Au thin film on an Al support by shearing a suspension of the particles between the round wire and the flat foil. (**I**) When the wire, with attached thin film and its Al support are suspended (attached to glass with red playdough) the soldered foil is strong enough to remain suspend and on the wire without delamination. The inset is a closer view of the joint showing that the particles are smear on the surface of the foil.

Manufacturing by means of undercooled particles could also be used for healing damaged surfaces, such as cracks, scratches, or other defects below the microscale as long as the surface bearing the defect can bond (chemical or mechanical) with the undercooled metal upon solidification. This approach can also be valuable in repairing delicate thin film materials where high temperature or large mechanical force cannot be applied. Figure 5A schematically shows an example of defect healing/repair where undercooled particles are used to fill a hole in a thin (200 nm) metal film formed on a flat substrate. Undercooled particles are drop-cast into the defect, then upon mechanical stressing, the stabilizing oxide layer is broken, allowing the liquid metal to flow and alloy with the film hence repairing the defect. Figure 5B shows the results of healing of damaged thin silver film (200 nm) using undercooled Field’s metal particles. For brevity, we refer to this type of joining as Heat-Free mechanical soldering (HFMS). Elemental maps (Fig. 5C) indicate that the damaged area on the film is healed with significant interpenetration by the components of the Field’s metal into the Ag film being repaired, suggesting that inter-diffusion and/or alloying enhances the nature of joint between the undercooled particles (heat-free ‘solder’) and the substrate. Compositional distribution of Field’s metal constituents, Bi, In, and Sn, is not homogeneous over the area covered, which probably stems from phase-segregation due to kinetic- and/or thermal- differentiation of the resultant alloy with Ag. In this study, we intentionally select healing materials that are compositionally different than the material being repaired to increase the contrast between phases, hence easier imaging, but healing/repairing can be achieved using undercooled particles from the same metal or its alloys.

Another example of the applications of HFMS is joining of thin films with concomitant delamination from the substrate to create a new composite material where the undercooled particles provide the inter-layer (Fig. 5D–G). To demonstrate this idea, a thin layer of Au (200 nm) is deposited on relatively thick aluminum foil and undercooled particles, sandwiched between layers of the Al/Au foil, are mechanically fractured (Fig. 5D). As a result, the Field’s metal not only ‘solders’ the two thin Au sheets together, but also delaminates it from the aluminum foil support indicating a stronger (thermodynamically favorable) interaction with Field’s metal over Al/Al_2_O_3_ (Fig. 5E–G). This results demonstrate that layered lamina material can be delaminated using HFMS where undercooled particles, specific to each layer, are used to induce delamination. Similarly, using less and/or smaller particles and tuning the applied stress as discussed above, many different joints could be obtained at ambient conditions. We used a high normal (orthogonal) stress in this example to allow for imaging, but rapid application of high shear stress gives the same HFMS effect albeit with a thinner inter-layer.

This study reports a technique in which undercooled particles are used for rapid joining and fabrication at ambient conditions. The capability of this technique are demonstrated with the formation of different shapes from spherical particle, discs and sheets, defect healing via HFMS. Applications of this technique, however, are not limited to these specific examples. To demonstrate versatility of our approach to make and use undercooled particles, we investigated eutectic bismuth-tin alloy, which has already been studied as a candidate of lead-free solders^30^,^36^,^65^. Even though BiSn at eutectic composition has much higher melting point (m.p. ≈ 139 °C) than Field’s metal (m.p. ≈ 62 °C), undercooled particles could be produced, at high yields, using SLICE (supporting information Figures S5 and S6). As observed with Field’s metal, undercooled particles of BiSn can be co-assembled with solidified ones without inducing solidification (supporting information Figure S5). When the particles solidify, as expected, spinodal decomposition of the eutectic occurs and can be observed in the EsB image (supporting information Figure S6).

## Conclusions

Stable undercooled particles of Field’s metal and eutectic Bi-Sn alloys are produced in high yields using SLICE. Stability of these meta-stable particles, we believe, is promoted by presence of protecting oxide-acetate layer. Gentle mechanical etching of outer layers by focused ion beam allows fabrication and joining of complex shapes. Depending on the state of the metal, ambient liquid or meta-stable liquid (undercooled), malleable or stiff structures with inherently different surface properties can be fabricated. For more robust macroscale applications, breaking of outer layers through mechanical stressing is demonstrated. Depending on the applied stress conditions, size of the particles, and the setup, we demonstrated healing of damaged surfaces and soldering/joining of metals at room temperatures without requiring high tech instrumentation, complex material preparation, or a high temperature process.

## Materials and Procedure

### Materials

Eutectic compositions of gallium – indium (Ga:In 75:25 wt%, m.p. ≈ 15.7 °C, Aldrich), bismuth – indium – tin (Field’s metal, Bi:In:Sn 32.5:51.0:16.5 wt%, m.p. ≈ 62 °C, Alfa Aesar) and bismuth – tin (Bi:Sn 58:42 wt%, m.p. ≈ 139 °C, Alfa Aesar) were used. For particle preparation, acetic acid (Biotech, sequencing grade), diethylene glycol (BioUltra) and ethanol (200 proof) were purchased from Fisher, Sigma, and Decon Laboratories Inc., respectively.

### Particle Preparation

The SLICE procedure^37^ was followed to form particles with metal core and oxide – acetate outer layer. 0.6 g (approx.) metal was added in acetic acid solution (5 vol% for Field’s metal, 1 vol% for Bi-Sn particles) in diethylene glycol. The solution prepared in a glass vial (scintillation vials, 20 mL) were kept in oil bath at determined temperature (120 °C for Field’s metal and 160 °C for Bi-Sn) for at least 2 min before subjected to shear to ensure metal melt. Shear was applied using a dremel 3000 variable speed rotary tool at the rate of 17,000 rpm with extender accessory and cross-shaped poly(tetrafluoroethylene) (PTFE) shearing implement. Shearing implement was placed as close as possible to vial wall to enhance the effect of shear. After 10 mins of continuous shearing, heat was withdrawn and the suspension was allowed to gradually cool, under fluidic shear for a further 5 minutes. Excess acetic acid and diethylene glycol were washed out with ethanol with the slurry resting on a filtering apparatus. Whatman #1 (particle retention of 11 μm), VWR Filter paper 494 (particle retention of 1 μm) and Whatman grade EPM 2000 (particle retention of 0.3 μm) filter papers were used for separation and cleaning of particles. Particles were stored in ethanol.

### Focused Ion Beam

Scanning electron microscopy-Focused Ion Beam (SEM-FIB): Zeiss NVision 40 Dual-Beam SEM-FIB was used to image the formed particles and mill away their surfaces. Imaging was performed at 2 kV with a working distance of 5.1 mm tilted at a 54° angle with a pixel size of 6.602 nm. Images were collected using the In-lens detector. A FIB of gallium ions was used to mill away a rectangular area directly over the formed EGaIn particles using an accelerating voltage of 30 kV and ion current of 1 pA. Milling was performed one frame at a time followed by imaging with the SEM.

### Healing of a surface damage

A 200 nm thick pure silver (99.99%) films were deposited on silicon wafer using e-beam evaporator (Temescal BJD-1800). Silver layer were manually damaged using a razor blade. Undercooled particles (>11 μm) in ethanol were drop casted into the defect on the silver surface and sheared using a glass rod or tube that was rolled over the defect. The surface was then template-stripped^45^,^66^ and surface characterized by SEM. For template stripping, a glass piece was cleaned with ethanol and dried with a stream of nitrogen gas. An estimated 5 μL of optical adhesive (Norland optical adhesive 61) was applied on a glass piece and glued on a substrate. The sample was exposed to UV light to cure the adhesive for 12 hours, then sample was lifted-off using a razor blade as previously described.

### Microsoldering

All manipulation experiments were performed under vacuum of 10^−6^ torr in a Zeiss NVision 40 FIB-SEM Dual Beam SEM. An Oxford Instruments Omniprobe system was used with a tungsten tip w/stainless steel shank that had a tip radius of 0.5 μm with a 8°–10° taper angle. The probe was used to pierce, spread, and collect undercooled particles and to join the tips of two probes. All movies are collected in real time.

### Heat-Free Mechanical Soldering

Aluminum foil (Reynolds Wrap) was deposited with a 200 nm thick gold (99.99%) film using the same e-beam evaporator system described above. Undercooled particles were sandwiched in between folded foil and sheared by side of a Pasteur pipette to weld two gold surfaces. Similar process is also employed using gold-coated needles (Signatone SE-TG, Gilroy, CA) to connect gold coated aluminum foil and the needle.

### Characterization

All metal particles were characterized with scanning electron microscopy (FEI Quanta 250 FE-SEM). The SEM were operated under high vacuum at the voltage of 8–10 kV. Both the secondary electron and the energy selective backscattering (EsB) mode were used to image the samples. Chemical characterization were conducted by energy dispersive X-Ray spectroscopy (EDS). Additional characterization was performed on a Zeiss Supra 55VP Field Emission SEM. Samples were imaged using an electron beam accelerating voltage of 3 kV and a working distance of 3.3 mm. Images were collected using an In-lens detector or an Everhart-Thornley secondary electron detector. Elemental analysis was performed at a working distance of 8.5 mm and using electron beam accelerating voltages of 15 kV. Elemental composition was determined using an Energy Dispersive x-ray Spectrometer with a silicon drift detector.

## Additional Information

**How to cite this article**: Çınar, S. *et al.* Mechanical Fracturing of Core-Shell Undercooled Metal Particles for Heat-Free Soldering. *Sci. Rep.* **6**, 21864; doi: 10.1038/srep21864 (2016).

## Supplementary Material

Supplementary Information

Supplementary Movie 1

Supplementary Movie 2
