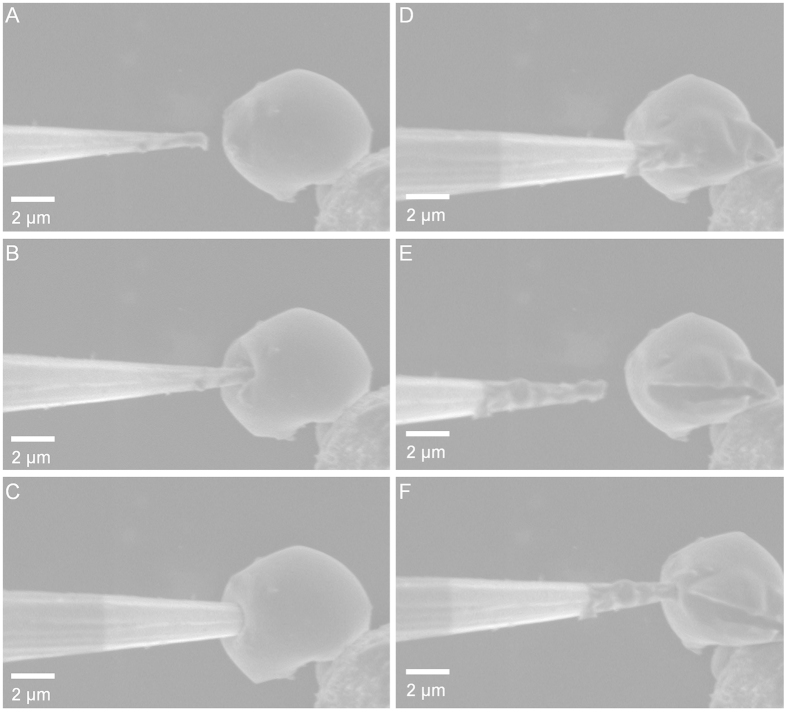# Corrigendum: Mechanical Fracturing of Core-Shell Undercooled Metal Particles for Heat-Free Soldering

**DOI:** 10.1038/srep25218

**Published:** 2016-05-04

**Authors:** Simge Çınar, Ian D. Tevis, Jiahao Chen, Martin Thuo

Scientific Reports 6: Article number: 2186410.1038/srep21864; published online: 02232016; updated: 05042016

In this Article, Figure 4 is incorrect. The Figure legend is correct. The correct Figure 4 appears below as Fig. 1.

## Figures and Tables

**Figure 1:**