# Supplementary material for: Mechanical Fracturing of Core-Shell Undercooled Metal Particles for Heat-Free Soldering
Source: Sci Rep. 2016 Feb 23;6:21864. doi: 10.1038/srep21864 (PMC4763186; doi:10.1038/srep21864)
Supplement: Supplementary Information [file srep21864-s1.pdf]

Mechanical Fracturing of Core-Shell Undercooled Metal Particles for Heat-Free Soldering

Supplementary Information

Simge Çınar, Ian D. Tevis, Jiahao Chen, Martin Thuo*

Department of Materials Science and Engineering, Iowa State University, Ames, IA 50011, USA

Microfabrication

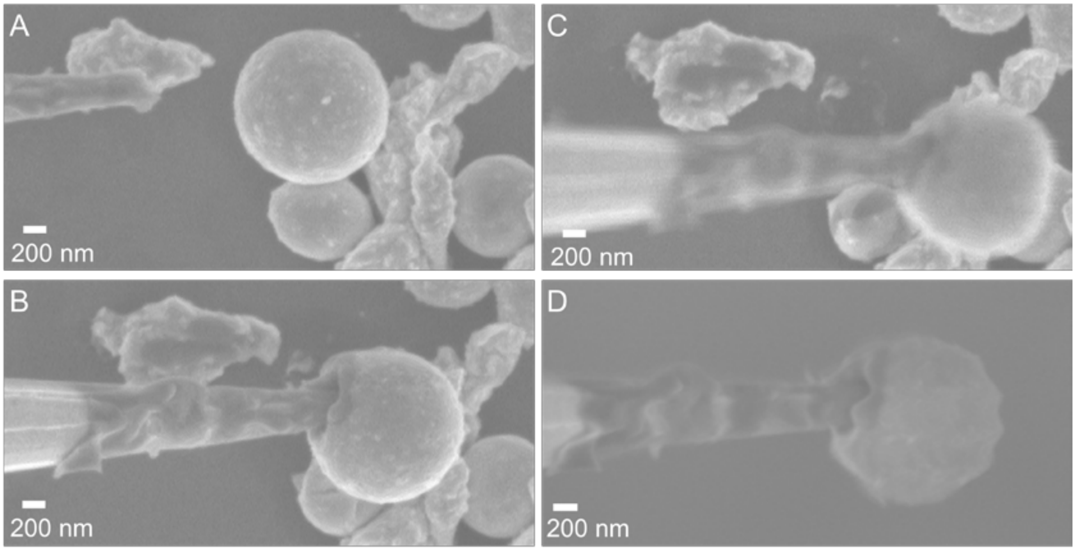

Figure S1: *A tungsten microprobe is used to penetrate and then solidify an undercooled Field's metal particle. A) A probe and particle before interacting. B) The probe is pushed into the liquid particle. C) The probe and particle are now joined together and are out of focus because they are being lifted from the surface. D) The probe and joined solid particle are now several millimeters above the surface and still joined together. The small waves in the image are caused by vibrations of the omniprobe because it is not touching the surface anymore.*

Macrofabrication:

In addition to high-level micro- and sub-microscale sculpturing described above, undercooled particles have an advantage of being convertible from liquids to solids at room temperatures in presence of nucleation/solidification catalyst sites. As an alternative to gentle removal of outer layer using FIB, the oxide layer can also be broken by applying external mechanical stress; that is, pressing the particles to fracture the outer layer, to induce flow followed by rapid coalescence and solidification. Figure 4A shows a micro-disk structure obtained after squishing the particles with a glass slide. Microstructure, as seen in the EsB detector image on the right (Figure S2A), and confirmed with an EDS map, shows microstructure due to phase-segregation analogous to those of the solid eutectic [1] indicating that the particles have solidified into a new shape. A similar process is applied to two adjacent assemblies of undercooled particles to give fused micro-disk dimers (Figure S2B) albeit with a thin boundary layer that could be due to trapped impurities or incomplete fusion of the flowing undercooled metal. In Figure S2C, we demonstrate that this method can be applied to make large flat areas (approx. 250 µm × 250 µm in this specific example), hence, sheet-like materials can be fabricated. If the particles are tightly packed in the assembly or molds are used to aid in compacting the liquid, then the method can be used to fabricate desired shapes/structures. Being able to manually smash many particles at the same time to give the same microstructure (Figure S2C) shows that almost all of the particles in the assembly are undercooled, and yield is close to unity (i.e. quantitative yield of undercooled particles). The eutectic-like microstructures and evenly distributed constituent elements (Supporting information Figure S2D) would result in near isotropic chemical and mechanical properties in the fabricated products.

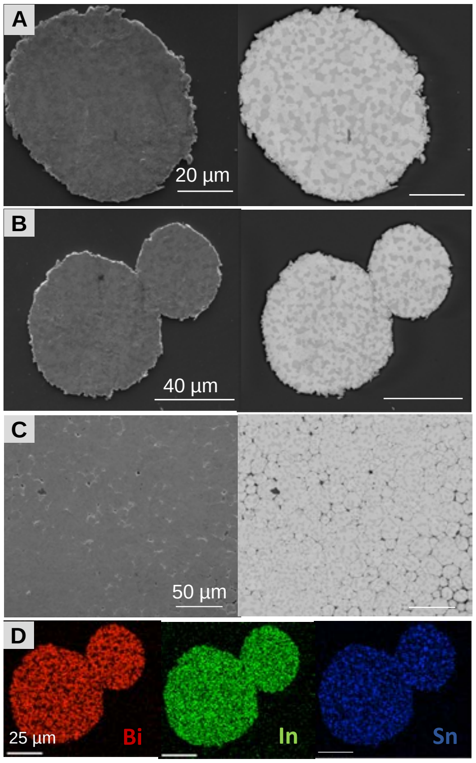

Figure S2: *Fabrication of simple disks, and related shapes from undercooled particles by mechanical fracture of outer layer (stress orthogonal to the plane of the particle monolayer). (A) A single disk, (B) a co-joined disk dimer, and (C) a sheet made from many particles assembled into a single layer. The SEM images, given on the left, compares the surface topology while the EsB detector images, given on the right, being sensitive to surface chemical composition, highlight surface phase-segregation of the eutectic metal upon solidification. (D) EDS mapping of dimers presented in (B) confirms eutectic compositions as shown by EsB detector. Elements are evenly distributed, with randomly distributed regions of slight enrichment, indicating the eutectic structure.*

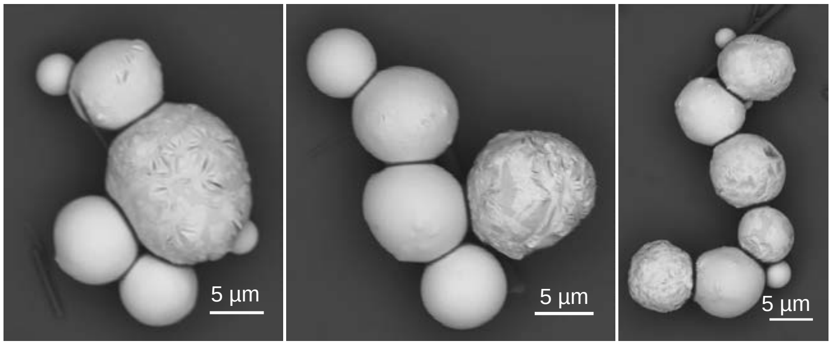

Figure S3: *EsB detector images of Field's metal particles. Majority of the particles had a smooth surface texture with few showing phase segregation.*

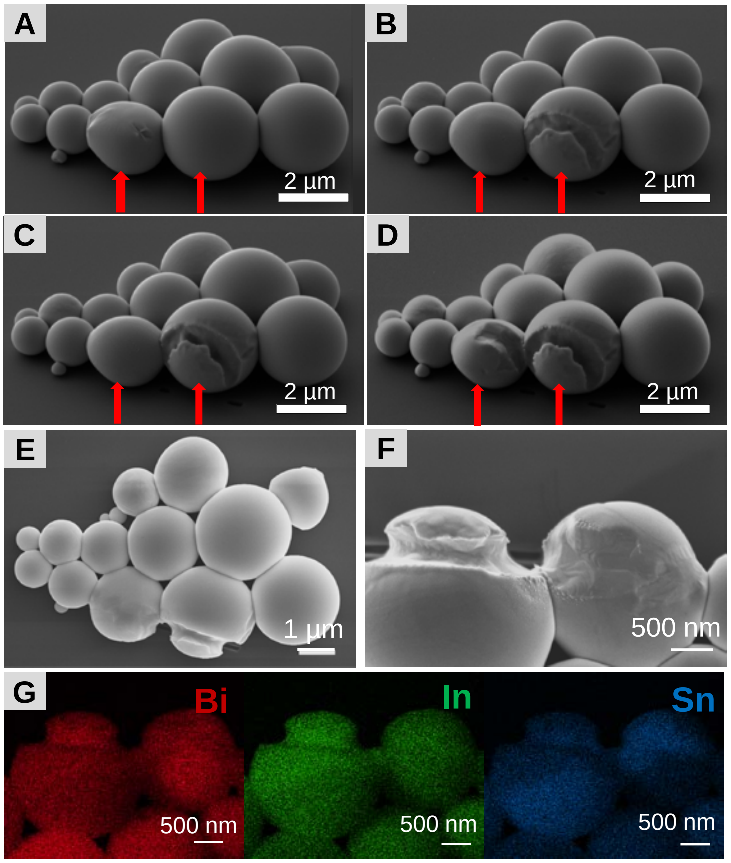

Figure S4: *Time course (from A to D) of a milling of an undercooled and a solidified particle by FIB. First solid particle is milled. Since it is solid, the particle is scratched. Then the undercooled particle next to it is milled. Because of its direct contact to the solid particle, undercooled particle is solidified as soon as the oxide layer is removed by milling. Solidification occurs very rapidly once there is a nucleation site, solid particle in this specific case. (E) and (F) are the top view of particles and the contact between solidified particles after being milled, respectively. (G) is the elemental EDS map of particles in (F), showing the distribution of Bi, In, and Sn.*

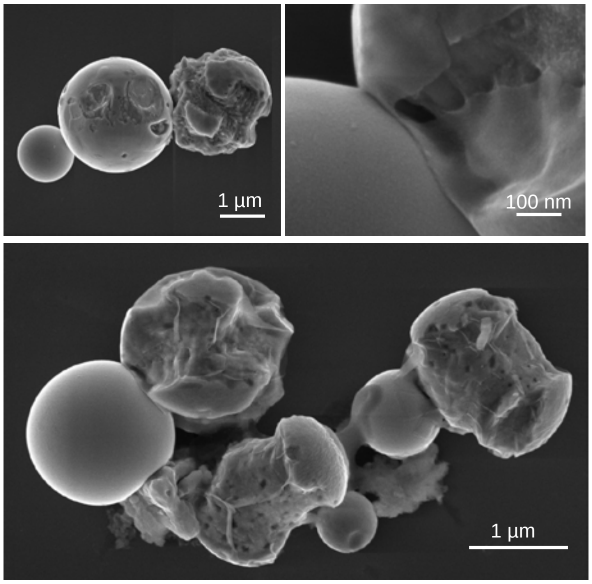

Figure S5: *Bismuth-tin particles. Direct contact of an undercooled particle to a rough surface do not induce solidification. Figure shows the stability of the undercooled particles encapsulated with oxide and acetate. Image also shows the undercooling of Bi-Sn particles produced by SLICE. Broken particles are probably due to the high thermal expansion coefficient difference between tin and bismuth. Bismuth expands during solidification while tin shrinks. During expansion of bismuth, tin-rich phases could be fallen of or separated from the original Bi-Sn particles.*

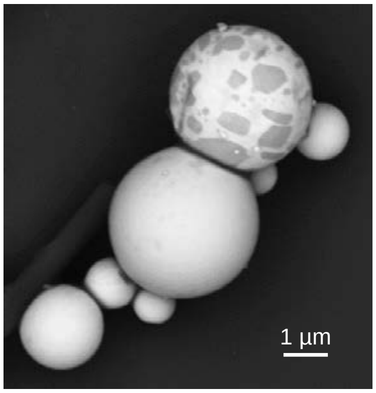

Figure S6: *Undercooled and phase segregated Bi-Sn particles produced by SLICE.*

Video S1: *A video derived from the sequence of events as given in Figure S4 indicating the solidification of a liquid particle upon milling and nucleation*

Video S2: *A video derived from the sequence of events as given in Figure S1 indicating the in situ modification of an SEM omniprobe by allowing a core-shell undercooled particle to solidify upon rupture of the shell layers.*

References

1. Çadırlı E, Böyük U, Kaya H, et al. The effect of growth rate on microstructure and microindentation hardness in the In–Bi–Sn ternary alloy at low melting point. *Journal of Alloys and Compounds*. 2009;470(1–2):150-156.
